# Role of CD133 antibody-conjugated nanocarrier in enhancing the targetability of hepatocellular carcinoma stem cells

**DOI:** 10.1038/s41598-025-14435-9

**Published:** 2025-08-19

**Authors:** Hadeer A. Aglan, Ahmed A. Abd-Rabou, Hanaa H. Ahmed, Ghada H. Elsayed, Mohamed S. Kishta, Manar A. Elhinnawi, Nadia S. Mahmoud

**Affiliations:** 1 Hormones Department, Medical Research and Clinical Studies Institute, National Research Centre, Giza, Egypt; 2 Stem Cell Laboratory, Center of Excellence for Advanced Sciences, National Research Centre, Giza, Egypt

**Keywords:** Hepatic cancer stem cells, CD133, PLGA nanoparticles, Quercetin, Kaempferol, Cancer, Nanoscience and technology

## Abstract

This study aimed at targeting hepatic cancer stem cells (CSCs) with quercetin (Q) or kaempferol (K) loaded into poly(lactide-co-glycolide) (PLGA) nanoparticles (NPs) decorated with CD133 antibody. For this purpose, the formulated Q NPs and K NPs and their free forms were evaluated for their cytotoxic potential, apoptotic activity, and anti-migratory effect against CD133^+^ CSCs isolated from the Huh7 cell line. Moreover, their influence on the hepatic CSCs-relevant molecular pathways was evaluated through analyzing several related gene expression levels. Interestingly, the in vitro study revealed that the Q NPs and K NPs and their free forms exhibit significant cytotoxic potential against CSCs isolated from the Huh7 cell line. The flow cytometric analysis revealed that Q NPs recorded the highest induction of apoptosis (77.8%) relative to the control (1.8%). The migration of hepatic CSCs is restrained by treatment with the suggested NPs and their free forms, but the most pronounced effect was observed after treatment with Q NPs. Both Q NPs and K NPs triggered significant down-regulation in the expression level of ABCG2, survivin, vimentin, cyclin D1, c-Myc, MMP-7, and VEGF genes in hepatic CSCs. The treatment with Q NPs motivated significant up-regulation in the expression level of the P53 gene in hepatic CSCs. Conclusively, the obtained results shed light on the success of Q NPs and K NPs modified with CD133 antibody on their surfaces in targeting hepatic CSCs. This effect was evidenced by their ability to significantly induce apoptosis, inhibit metastasis, reverse drug resistance, and interfere with CSC-associated signaling pathways.

## Introduction

Liver cancer is the 3^rd^ leading cause of cancer-related deaths globally, with hepatocellular carcinoma (HCC) accounting for the majority of cases^1^. The incidence of HCC is rising by 2–4% annually across the global population and is projected to surpass one million cases per year by 2025^2^. Similarly, Egypt has witnessed a nearly twofold increase in HCC cases over the last decade^3^. In Egypt, HCC is the primary cause of cancer-related morbidity and mortality and is the most prevalent cancer among males^4^. The average age of HCC onset in Egypt is 58 years, compared to 46 years in other countries^5^.

Surgery is the therapeutic choice for a limited number of patients with HCC, while the majority undergo locoregional treatments, including percutaneous ethanol injection, radiofrequency ablation, transarterial chemoembolization (TACE), radioembolization, radiotherapy, or transarterial radioembolization. Systemic treatment is typically reserved for patients who are ineligible for locoregional therapies or do not respond to TACE. Recently, the introduction of novel immunotherapies, such as immune checkpoint inhibitors, has started to transform the landscape of systemic HCC therapy^6^.

For years, liver cancer stem cells (CSCs) have been extensively studied as a key factor contributing to poor treatment outcomes and tumor relapse in liver cancer patients. CSCs, also recognized as tumor-initiating cells (TICs), constitute malignant cells within the tumor that retain self-renewal and differentiation capabilities, enabling them to drive tumorigenesis. These cells play a crucial role in therapeutic resistance, as they exhibit high resistance to conventional chemotherapy, evade apoptosis, survive treatment, and repopulate the tumor with limited proliferative potential. Additionally, CSCs can metastasize, leading to the formation of new tumors^7^.

Xenotransplantation and fluorescence-activated cell sorting (FACS) experiments have identified key indicators for CSCs in HCC, which can be categorized into intracellular indicators, like cytokeratin 19 (CK19), and surface indicators, such as CD13, CD24, CD44, CD47, CD90, CD133, and epithelial cellular adhesion molecule (EpCAM)^8^. Among these, CD133 is a widely recognized transmembrane glycoprotein and a universal CSC indicator over numerous cancers^9^. In HCC, CD133 is highly expressed in CSCs^10^ and has been recognized as a crucial target for enhancing the effectiveness of chemotherapy in recurrent HCC cases^11^. CD133-expressing HCC stem cells are also implicated in liver tumorigenicity and resistance to radiotherapy, primarily due to the activation of key signaling pathways, including AKT/Protein kinase B (PKB), B-cell lymphoma 2 (Bcl-2)^12^, and mitogen-activated protein kinase (MAPK)/PI3K^13^. Additionally, CSCs and normal stem cells engage many essential stemness-related signaling pathways, like Wnt/β-Catenin, Notch, nuclear factor kappa B (NF-κB), Hedgehog (HH), and Janus kinase/signal transducer and activator (JAK/STAT) transcription proteins in HCC^14^. Therefore, monitoring CSCs’ progression and regression is crucial for improving the diagnosis and treatment of HCC^15^.

Nanotechnology offers a promising and innovative approach for combating cancer, especially in targeting the evasive and treatment-resistant CSCs^16^. There is growing interest in utilizing nanoformulation-based anticancer medications, where NPs are particularly designed to recognize and target CSCs based on their molecular markers^17^. The NPs exhibit superior encapsulation capabilities, lack heterogeneity in tumors, and leverage the enhanced permeability and retention (EPR) effect, allowing for better accumulation within tumor regions. As a result, NP-based targeted therapy enhances therapeutic efficacy while reducing off-target effects^18^. NP delivery systems can be classified into passive and active targeting strategies. Passive targeting relies on the natural accumulation of NPs in tumor sites through the EPR effect^19^, but its effectiveness is limited due to tumor heterogeneity and insufficient accumulation^20^. To overcome these challenges, active targeting enhances the EPR effect and improves drug delivery efficiency beyond passive targeting alone^21^. This targeting is achieved by functionalizing the NP surface with ligands that facilitate affinity recognition, retention, and uptake by target cells^22^. This approach relies on specific intermolecular recognition^23^, where ligand-receptor interactions on target cell surfaces increase NP uptake, ultimately enhancing therapeutic efficacy^24^.

Polymeric nanoparticles are widely regarded as the most effective drug delivery vehicles due to their exceptional pharmacokinetic properties. They offer several advantages over conventional drug delivery systems, including high durability, compatibility with both hydrophobic and hydrophilic substances, strong resistance to degradation by enzymes, reduced frequency of drug administration, and, most importantly, minimized harmful side effects^25^. To specifically target and eliminate CSCs, CSC-specific antibodies or ligands can be conjugated onto the surface or interior of NPs^26^. A notable advancement in this field is the evolution of CD133-targeted NPs, developed to selectively deliver therapeutic candidates to cancer cells expressing the CD133 antigen. This targeting is fulfilled by modifying NP surfaces with antibodies, peptides, or small molecules that exhibit high affinity for CD133, ensuring precise recognition of CD133-expressing CSCs^27^. Studies have demonstrated that NPs functionalized with anti-CD133 antibodies and loaded with chemotherapeutic agents can effectively target CD133-positive CSCs, leading to tumor outgrowth inhibition in the liver cancer and glioblastoma mouse models^28^.

Natural compounds have long served as a valuable source of biologically active molecules capable of interacting with numerous cellular targets while minimizing the adverse side events usually linked with cancer treatments^29^. One such compound is quercetin (Q), a flavonoid with the chemical structure 3,3’,4’,5,7-pentahydroxyflavone^30^. It exhibits a broad range of biological functions, making it a hopeful candidate for cancer therapy. Its mode of action involves tumor cell cycle suppression, apoptosis induction, ROS modulation, and the reduction of chemotherapeutic resistance. Given these activities, pharmaceutical engineering has focused on developing advanced nanocarriers to enhance quercetin’s bioavailability and targeting capabilities, thereby improving its therapeutic efficiency against HCC and addressing anticancer drug resistance^31^. Ren et al.^32^ successfully synthesized Q NPs by utilizing gold NPs, Q solution, and poly (DL-lactide-co-glycolide) (PLGA) for liver cancer treatment. These Q NPs effectively inhibited cell growth of liver cancer and colony formation by upregulating p27 and downregulating c-Myc, cyclinD1, cyclin-dependent kinase 1 (CDK1), matrix metallopeptidase 7, and β-catenin within the cancer cells. These findings suggest that the Q NPs have significant potential as a multifunctional candidate for liver cancer therapy.

Kaempferol (K) (3,5,7-trihydroxy-2-(4-hydroxyphenyl)-4 H-chromen-4-one) is a flavonoid abundantly present in fruits and vegetables^33^, including onions, green tea, grapes, potatoes, tomatoes, apples, cucumbers, broccoli, blackberries, and green beans^34^. It functions as a free radical scavenger and supports the activity of several antioxidant enzymes. K exerts its chemopreventive effects through multiple mechanisms, including cell cycle arrest, anti-proliferation, anti-angiogenesis, and apoptosis induction^35^. Despite its extensive pharmacological properties, the biomedical application of K is limited due to its poor water solubility, low permeability, instability in alkaline aqueous environments, extensive metabolic processing before systemic absorption, and poor oral bioavailability^36^. A promising approach to overcoming these limitations is the evolution of NPs-based drug delivery systems, which enhance the oral bioavailability of hydrophobic and lipophilic compounds like K^37^ due to their high biocompatibility^38^. Mechanistic studies have demonstrated that K-conjugated NPs promote oxidative stress-mediated apoptosis and cell cycle arrest in liver cancer cells, highlighting their potential as an anticancer therapy^33^. For instance, K-coated silver NPs displayed a synergistic apoptotic effect in liver cancer (HepG2) cells. This was characterized by a reduction in B-cell leukemia/lymphoma 2 (Bcl-2) protein levels, an increase in Bcl-2-associated protein x (Bax) and cytochrome C levels, caspase-3 activation owing to mitochondrial membrane disruption, and enhanced p53-mediated cell cycle arrest. These findings underscore the potential of K-based nanotherapeutics in liver cancer treatment^39^.

Therefore, utilizing the unique advantages of nanotechnology for enhancing the therapeutic efficacy of HCC heralds a new era of precision medicine. Thus, this research underscores the potential of NPs-based technology in improving HCC treatment outcomes with special emphasis on the role of CD133-antibody conjugated PLGA NPs to selectively target and deliver Q or K to CSCs in the Huh7 cell line. The successful treatment of CSCs *via* influencing numerous cellular mechanisms simultaneously would prohibit the survival of this evasive subpopulation and consequently hepatic cancer metastasis, relapse, and drug resistance.

## Materials and methods

### Materials

Quercetin (Q), kaempferol (K), dichloromethane (DCM), poly(lactide-co-glycolide) (PLGA), polyvinyl alcohol (PVA), bovine serum albumin (BSA), 3-[4,5-dimethylthiazol-2-yl]-2,5 diphenyl tetrazolium bromide (MTT), dimethyl sulfoxide (DMSO), 1-ethyl-3-(3-dimethylaminopropyl)carbodiimide (EDC) and N-hydroxysuccinimide (NHS) were purchased from Sigma-Aldrich (USA). Regenerated cellulose membranes (Amicon 10,000 MWCO ultrafilter) and dialysis bag (MW cut-off 10 kDa) were supplied from Millipore (USA). Dulbecco’s Modified Eagle’s Medium (DMEM), fetal bovine serum (FBS), penicillin-streptomycin, and phosphate-buffered saline (PBS) were procured from Biowest (France). CD133 MicroBeads kit, LS MACS column, phycoerythrin (PE)-conjugated CD24 and CD133 antibodies, as well as fluorescein isothiocyanate (FITC)-conjugated CD34 and CD326 antibodies, were obtained from Miltenyi Biotec. (Germany). Additionally, PE-conjugated CD44 and CD90 antibodies were purchased from R&D Systems, Abingdon (UK).

### Methods

#### I. Nanoparticles Preparation and characterization

##### Preparation of nanoparticles

Q- and K-loaded PLGA nanoparticles (non-targeted) were prepared using the emulsification-solvent evaporation method, with slight modifications based on previously reported protocols^40^. Briefly, 10 mg of Q (quercetin) and 10 mg of K (kaempferol) were dissolved in 5 mL of dichloromethane (DCM) containing 100 mg of PLGA (50:50, Mw 30,000 Da), yielding a drug-to-polymer ratio of 1:10 (w/w). This organic phase was slowly added dropwise into 20 mL of an aqueous solution of 1% (w/v) polyvinyl alcohol (PVA; 89% hydrolyzed, Mw 30,000 Da) under continuous stirring. The resulting mixture was sonicated using a probe sonicator (e.g., Vibra-Cell VCX130, Sonics & Materials Inc., USA) at 40% amplitude for 2 min in an ice bath to form a stable oil-in-water (O/W) emulsion. To evaporate the organic solvent (DCM), the emulsion was stirred at 500 rpm at room temperature for 4 h on a magnetic stirrer. Following solvent evaporation, nanoparticles were recovered by centrifugation at 20,000 rpm for 30 min at 4 °C (using a high-speed centrifuge such as Beckman Coulter Avanti J-26 XPI). The pellets were washed three times with distilled water to remove residual PVA and unencapsulated drug. The purified nanoparticles were then lyophilized (Labconco FreeZone 2.5) and stored at 4 °C in airtight containers for further analysis.

##### Decoration of nanoparticles with CD133 antibody (targeted)

For active targeting, CD133 antibodies were covalently conjugated to the surface of PLGA nanoparticles using EDC/NHS chemistry. Lyophilized nanoparticles were first dispersed in 10 mL of PBS (pH 7.4), followed by the addition of 10 mg of EDC and 5 mg of NHS. The mixture was stirred at room temperature for 4 h to activate the carboxyl groups on the PLGA surface. Activated nanoparticles were then separated by ultracentrifugation at 25,000 rpm for 30 min at 4 °C to remove unreacted EDC and NHS. The pellet was resuspended in 5 mL PBS, and 50 µg of CD133 antibody was added. The conjugation mixture was stirred for 2 h at room temperature, followed by overnight incubation at 4 °C. On the following day, ultracentrifugation was repeated at 25,000 rpm for 30 min at 4 °C to remove unconjugated antibodies. The supernatant was collected, and unconjugated antibody was quantified using the bicinchoninic acid (BCA) protein assay (Thermo Fisher Scientific, USA) according to the manufacturer’s instructions. The pellet (antibody-conjugated NPs) was lyophilized and stored at 4 °C. For control experiments, bovine serum albumin (BSA)-decorated nanoparticles were synthesized using the same conjugation protocol, replacing the CD133 antibody with BSA^40^.

##### Nanoparticles characterization

- Transmission electron microscope (TEM).

The morphology of the NPs was analyzed using TEM (JEOL JEM-1400). A 100 µg/mL suspension of the NPs was deposited onto Formvar-coated copper grids and allowed to dry completely at room temperature. Once dried, imaging and structural analysis were performed using Digital Micrograph and Soft Imaging Viewer Software, enabling detailed visualization of the NP size, shape, and surface characteristics.

- Particle size distribution and zeta potential.

The dynamic particle size and surface charge (zeta potential) of the prepared NPs were determined using a Dynamic Light Scattering (DLS) instrument (Zetasizer Nano ZS, Malvern Instruments, UK) equipped with a 633 nm laser. To ensure instrument accuracy, a reference standard (DTS1230, zeta-potential standard from Malvern) was used for calibration. For measurements, 1 mL of the NP suspension was placed in a disposable transparent sizing cuvette. Data analysis was performed using Malvern Instruments’ Dispersion Technology Software (Version 4.0). The values of zeta-potential were calculated from the measured electrophoretic mobility data using the Smoluchowski equation.

- Fourier transform infrared spectroscopy (FTIR).

The functional groups of the developed NPs were analyzed using FTIR spectroscopy (Bruker Vertex 80v). The spectra were recorded over a wavelength range of 4000–400 cm⁻¹ with high resolution to identify characteristic functional group vibrations and confirm successful NP formulation and surface modifications.

- Entrapment efficiency measurement.

The entrapment efficiency (EE%) of the NPs was detected by calculating the ratio of the incorporated Q and K to the total amount initially added during formulation according to Eq. (1). To remove impurities and free, non-conjugated compounds, the prepared nanoformulations underwent purification using the dialysis tubing technique. This process involved elution through regenerated cellulose membranes (Amicon 10,000 MWCO ultrafilter). The quantification of encapsulated compounds was performed using a microplate reader (BMG Labtech, Germany), with all data processed against calibration curves specific to each compound.

1 $${\text{Entrapment Efficiency }}\left( {{\text{EE\% }}} \right){\text{ }}=({\text{Amount of drug encapsulated in nanoparticles}}\, \div \,{\text{Total amount of drug initially added}}){\text{ }} \times {\text{ 1}}00$$

- Drug release profiles.

To evaluate the drug release profile, a specific amount of each nanoformulation and its corresponding free form were diffused in a freshly prepared release medium inside a dialysis bag (MW cut-off 10 kDa). The sealed dialysis bag was then immersed in 30 mL of release medium (pH 7.4) and placed in a shaking incubator at 37 °C under mild stirring to simulate physiological conditions. At predetermined time intervals (0, 1, 2, 3, 4, 5, and 24 h), 1 mL of the release medium was withdrawn and replaced with an equal volume of fresh medium to maintain sink conditions. The collected samples were filtered, and the concentration of the released drug was quantified using a microplate reader (BMG Labtech, Germany)^41,42^. Furthermore, drug release kinetic analysis was performed using mathematical relations^43,44^.

#### II. Cancer stem cells isolation and characterization

##### Cell line

The Huh7 cell line was purchased from VACSERA Co. (Egypt), which obtained it from the American Type Culture Collection Center. The cells were cultured in DMEM supplemented with 10% FBS and 100 IU/mL penicillin G − 100 µg/mL streptomycin. The cells were maintained at 37 °C in a humidified incubator with 5% CO₂ to ensure optimal growth conditions.

##### Isolation and culture of hepatic cancer stem cells (CSCs)

CD133⁺ hepatic CSCs were isolated from the Huh7 cell line using the CD133 MicroBeads kit following the manufacturer’s protocol. The cells were magnetically labeled with CD133 MicroBeads – Tumor Tissue. Thereafter, the labeled cell suspension was loaded onto an LS MACS column placed within a MidiMACS™ Separator (Miltenyi Biotec). The magnetically labeled CD133⁺ cells were retained in the column, while the unlabeled cells passed through, depleting the fraction of CD133⁺ cells. After removing the column from the magnetic field, the retained CD133⁺ cells were eluted as the positively selected fraction. To enhance purity, the positively selected fraction was subjected to a second round of separation using a new column.

##### Characterization of the isolated cells

- Flow cytometric analysis.

To confirm that the isolated cells were CSCs, they were characterized using flow cytometric analysis for specific surface markers, including CD24, CD34, CD44, CD90, CD133, and CD326 (EpCAM). For staining, PE-conjugated CD24, CD44, CD90 and CD133 antibodies, as well as FITC-conjugated CD34 and CD326 antibodies were used. The cells were incubated with each antibody for 20 min in the dark at room temperature, following the manufacturer’s recommended concentrations. The stained cells were then analyzed using a Beckman Coulter Elite XL flow cytometer (California, USA) to confirm the expression of CSC surface markers, ensuring the successful isolation of hepatic CSCs^12^.

- Real time PCR analysis.

RNA was extracted from the isolated CSCs using the RNeasy Mini Kit (Cat#74104, Qiagen, Germany) following the manufacturer’s instructions. The concentration and purity of the extracted RNA were assessed using a NanoDrop 2000 spectrophotometer (Thermo Fisher Scientific, Rockford, IL, USA), with the 260/280 nm absorbance ratio used to determine RNA quality. Complementary DNA (cDNA) synthesis was performed using the RevertAid First Strand cDNA Synthesis Kit (Cat# K1621, Thermo Fisher Scientific, Inc., Lithuania) according to the manufacturer’s protocol. For gene expression analysis, quantitative real-time PCR (qRT-PCR) was carried out using the DNA-Technology Real-Time PCR device (DT lite 4, Russia) to evaluate the expression levels of NOTCH1, NOTCH2, NOTCH3, and aldehyde dehydrogenase 1 A1 (ALDH1A1). The reaction mixture (25 µl volume) contained 12.5 µl of QuantiTect SYBR Green master mix (Cat# 204141, Qiagen, Germany), 0.75 µl of forward and reverse primer of target gene (Invitrogen, USA), 1.5 µl cDNA template and 9.5 µl RNase free water. β-actin was used as a housekeeping gene. Relative mRNA expression *versus* control value was assessed using the 2^−ΔΔCt^ comparative method after normalization with β-actin gene.

The PCR cycling was set as follows: initial denaturation step at 95 °C for 15 min, followed by 40 cycles of denaturation at 94 °C for 15 s, annealing at 53 °C for NOTCH1 gene, 55 °C for NOTCH2 gene, 60 °C for NOTCH3 and ALDH1A1 genes for 30 s, and extension at 72 °C for 30 s. The primer sequences of each target gene are delineated in Table 1.

**Table 1:** List of gene-specific primers in RT-PCR.

Gene	Forward	Reverse	Ref
NOTCH1	CCCGCCAGAGTGGACAGGTCAGTA	TGTCGCAGTTGGAGCCCTCGTTA	^45^
NOTCH2	CCCACAATGGACAGGACA	GAGGCGAAGGCACAATCA	
NOTCH3	TCTCAGACTGGTCCGAATCCAC	CCAAGATCTAAGAACTGACGAGCG	
ALDH1A1	TTACCTGTCCTACTCACGATT	GCCTTGTCAACATCCTCCTTAT	^46^
β-actin	AGAGCTACGAGCTGCCTGAC	AGCACTGTGTTGGCGTACAG	^47^

- Chemotherapy sensitivity assays.

The sensitivities of the isolated CSCs to chemotherapeutic drugs were evaluated utilizing the Cell Counting Kit-8 (CCK-8) assay (Sigma-Aldrich, USA), which is based on the WST-8 [2-(2-methoxy-4-nitrophenyl)-3-(4-nitrophenyl)-5-(2,4-disulfophenyl)-2 H tetrazolium] colorimetric method. Briefly, 4000 cells/well were seeded in 96-well plates, and various concentrations (0.01, 0.1, 1, 10, and 100 µg/ml) of doxorubicin (Adricin, Doxorubicin Hcl, Hikma Specialized Pharmaceuticals, Egypt) were added at the beginning. After 72 h incubation, viable cells were measured by CCK-8 assay following the manufacturer’s instructions at 450 nm, using a microplate reader (Model 500; BIORed Instrument Inc., USA)^48^.

#### III. Evaluation of the anti-tumor efficacy of Q or K-loaded PLGA NPs with CD133 antibody against hepatic CSCs

##### Cytotoxic assay

The 3-[4,5-dimethylthiazol-2-yl]-2,5 diphenyl tetrazolium bromide is based on the conversion of MTT into formazan crystals by living cells, which reflects cytotoxicity based on mitochondrial activity^49^. The cytotoxic impact of the formulated NPs with CD133 antibody and loaded with the suggested natural compounds (Q or K) *versus* their free forms was measured by MTT assay using CSCs isolated from the Huh7 cell line. The cells were incubated with different concentrations of the compounds (20, 40, 80, and 160 µg/ml) for 24 h and 72 h at a cell density of 1 × 10^4^ cells/well of a 96-well plate. After the different incubation time, MTT dissolved in PBS was added to each well at a final concentration of 5 mg/ml, and the samples were incubated at 37 °C for 4 h. After 4 hours, the medium was decanted and dimethyl sulfoxide (DMSO) was added to each well, including blank wells and left for 30 min to dissolve formazan crystals that formed during MTT cleavage in actively metabolizing cells. Absorbance of formazan in each plate was measured at 492 nm, using a microplate reader (Model 500; BIORad Instrument Inc., USA). For the untreated cells (negative control), medium was added instead of the test compound. All tests and analyses were done in triplicate, and the results were averaged.

##### Apoptotic assay

Annexin V and propidium iodide (PI) were applied for the determination of CSCs death mechanistic approach after treatments with the formulated NPs conjugated with CD133 antibody and loaded with Q or K and their free counterparts at 24 h of drug incubation. CSCs stained with only annexin V, only PI, or both dyes were considered as early apoptotic, necrotic, or late apoptotic cells, respectively. Therefore, the apoptotic analysis was dedicated to differentiating between early and late apoptotic cells, as well as necrotic cells. The apoptosis of the treated and untreated control cells was analyzed by flow cytometer (Beckman 230 Coulter Instrument, USA)^50^.

##### Metastatic (in vitro scratch) assay

In vitro scratch or wound-healing assay was used to assess the activity of the formulated NPs with CD133 antibody and loaded with Q or K and their free forms on the migration of hepatic CSCs. Cells were seeded in 12-well plates (2 × 10^5^/well) with complete medium overnight to obtain a full confluent monolayer. After 24 h, the complete medium was removed and the confluent cell sheet was wounded through scratching the culture well surface with a 200 µl pipette tip. The scratch-wounded cells were washed twice with PBS to remove any cell fragments or detached cells before incubating in fresh medium with the tested compounds in either nanoforms or free forms for 24 h. Cell migration was monitored, and images of wound healing were captured by using the microscope after 24 h^51^.

##### Gene expression analysis

CSCs were seeded at a cell density of 4 × 10^4^ cells/well and were treated with Q or K nanoformulations or their free forms for 48 h. Total RNA was isolated from cell samples using the RNAeasy mini Kit (Qiagen, Germany) then the concentration and purity of total extracted RNA were determined using a NanoDrop UV spectrophotometer (Thermo Fisher Scientific, USA). RNA of each treatment was converted to first-strand cDNA according to the manufacturer’s recommendations using Revert Aid First Strand cDNA Synthesis Kit (Thermo Fisher Scientific, Lithuania). Specific primer sequences (Table 2) for the drug resistance-related gene ATP-binding cassette superfamily G member 2 (ABCG2), P53, survivin, cyclin D1, c-Myc, vimentin, vascular endothelial growth factor (VEGF), and matrix metalloproteinase 7 (MMP-7) were used for quantitative real-time PCR. The reaction mixture (25 µl volume) included 12.5 µl of Maxima SYBR Green qPCR master mix (ThermoScientific, Lithuania), 0.75 µl of forward and reverse primers of the target gene (Invitrogen, USA), 1.5 µl cDNA template, and 9.5 µl RNase-free water. β-actin was used as a housekeeping gene. Relative mRNA expression *versus* control value was assessed using the 2^−ΔΔCt^ comparative method after normalization with the β-actin gene.

The PCR cycling was set as follows: initial denaturation step at 95 °C for 15 min, followed by 40 cycles of denaturation at 94 °C for 15 s, annealing at 55 °C for 30 s, and extension at 72 °C for 30 s. DNA Technology Detecting Thermocycler DT Lite 4S1 (Russia) was used for gene expression quantitation.

**Table 2:** Sequences of the primers used in the RT- PCR analysis.

Gene	Forward primer (5′-3′)	Reverse primer (5′-3′)	Ref.
ABCG2	TATAGCTCAGATCATTGTCACAGTC	GTTGGTCGTCAGGAAGAAGAG	^52^
P53	TCAACAAGATGTTTTGCCAACTG	ATGTGCTGTGACTGCTTGTAGATG	^53^
Survivin	GGACCACCGCATCTCTACAT	GCACTTTCTTCGCAGTTTCC	^54^
Cyclin D1	GCCAGAGGCGGAGGAGAACA	AAGCGTGTGAGGCGGTAGTA	^55^
c-Myc	AGAGAAGCTGGCCTCCTACC	CGTCGAGGAGAGCAGAGAAT	
Vimentin	GTGGAGCGCGACAACCTG	GACGTGCCAGAGACGCATTG	^56^
VEGF	TCGGGCCTCCGAAACCATGA	CCTGGTGAGAGATCTGGTTC	^57^
MMP-7	GTGGTCACCTACAGGATCGTA	CTGAAGTTTCTATTTCTTTCTTGA	^58^

### Statistical analysis

The obtained results are represented by the means ± standard deviations (SD). Statistical analysis was performed using one-way analysis of variance (ANOVA) with the Statistical Package for the Social Sciences (SPSS), version 14. Post-hoc comparisons between groups were conducted using the least significant difference (LSD) test. A P-value < 0.05 was considered statistically significant.

## Results

### Characterization of the formulated NPs

#### Morphology and size of the prepared NPs using TEM

**Fig. 1:**
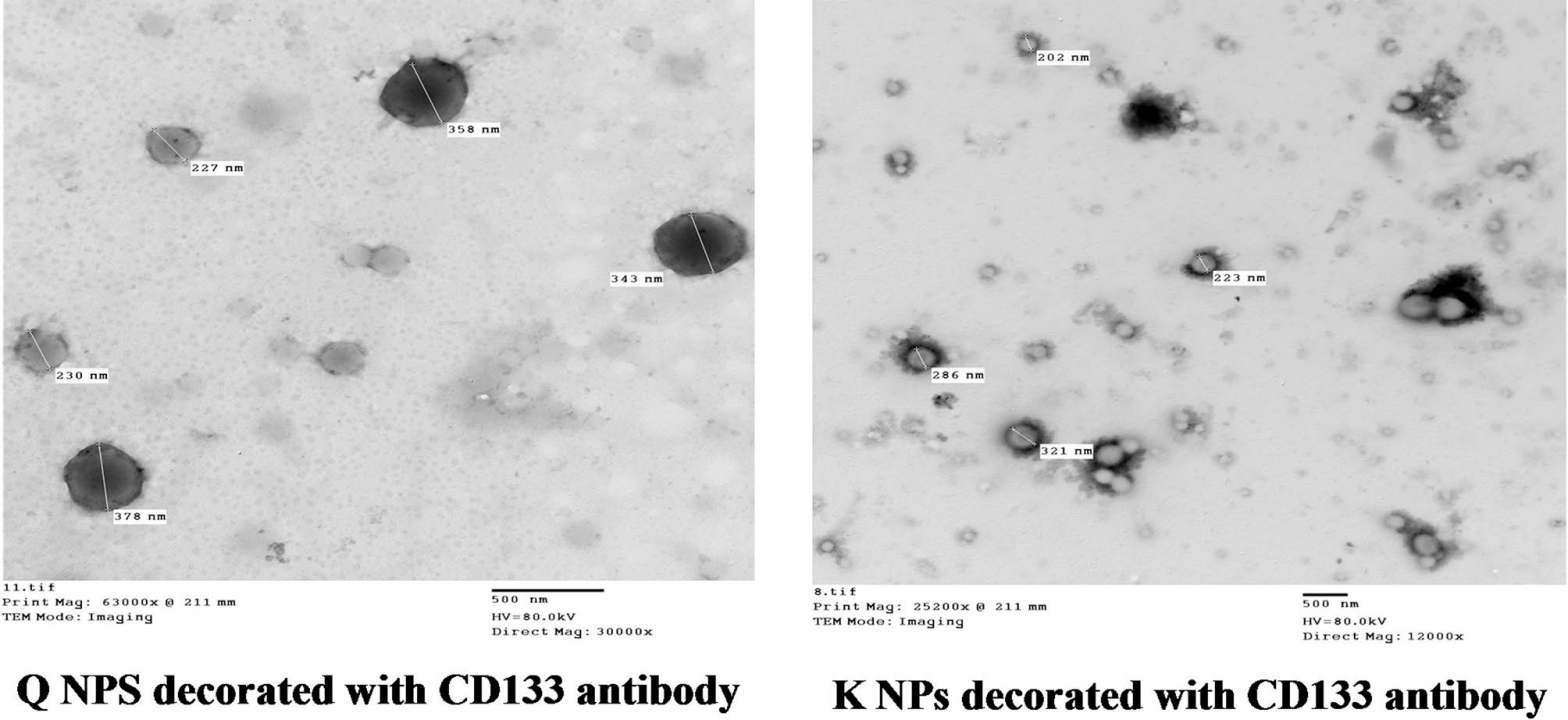
Transmission electron microscope images of the prepared nanoparticles.

TEM images revealed that the synthesized NPs were spherical and conjugated with the CD133 antibody (Fig. 1). The average particle size of Q NPs with CD133 antibody ranged from 227 nm to 378 nm, whereas the K NPs with CD133 antibody exhibited an average size range of 202 nm to 321 nm.

##### Particle size distribution and zeta potential of the prepared NPs

**Fig. 2:**
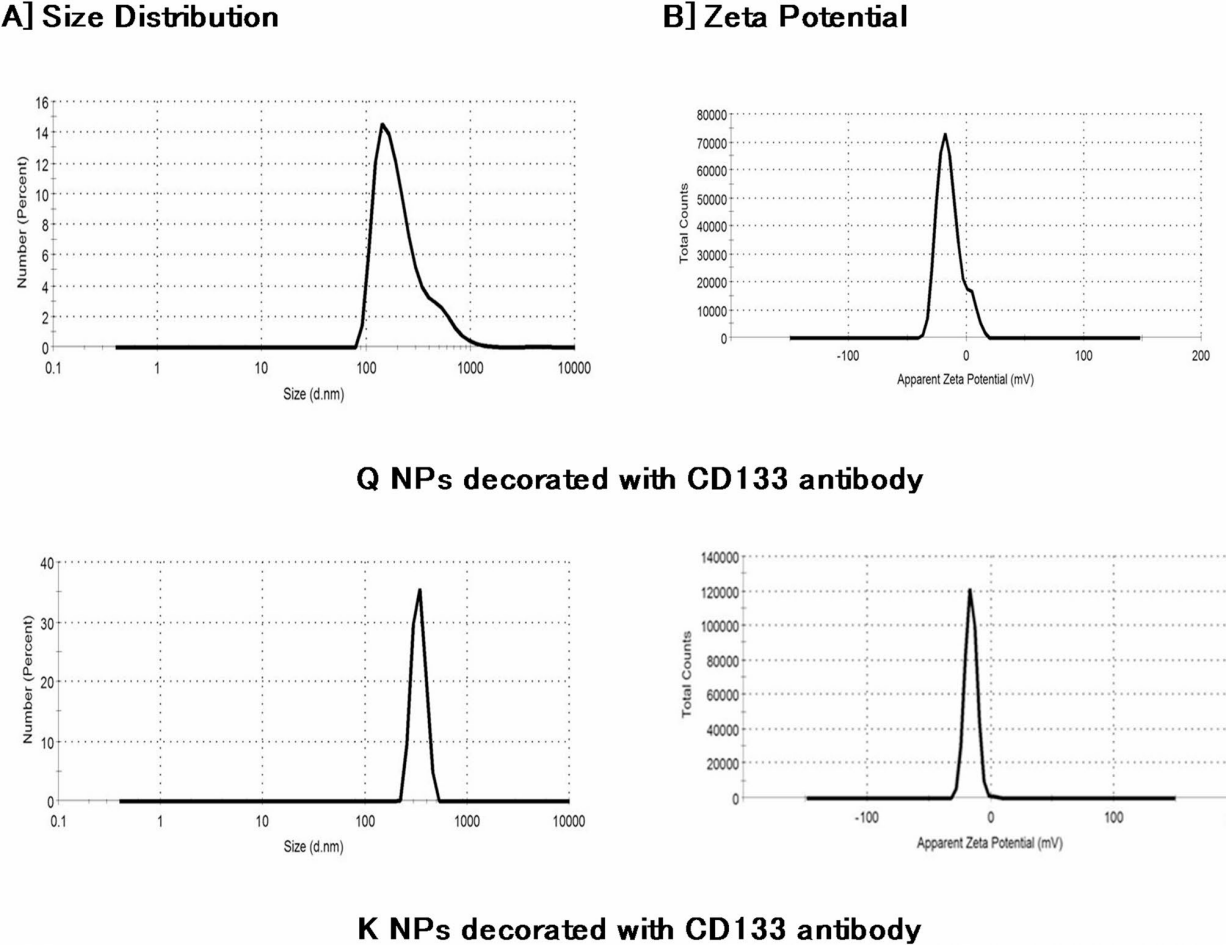
Particle size distribution and zeta potential charges of the prepared nanoparticles.

The particle size distribution and zeta potential of the synthesized NPs decorated with CD133 antibody were analyzed using a dynamic light scattering (DLS) instrument **(**Fig. 2**)**. The Q NPs exhibited an average dynamic particle size of 234.8 nm and a negatively charged zeta potential of -14.5 mV. In comparison, the K NPs showed a larger average size of 336.4 nm and a zeta potential of -15.9 mV.

##### Fourier transform infrared spectroscopy (FTIR) analysis

**Fig. 3:**
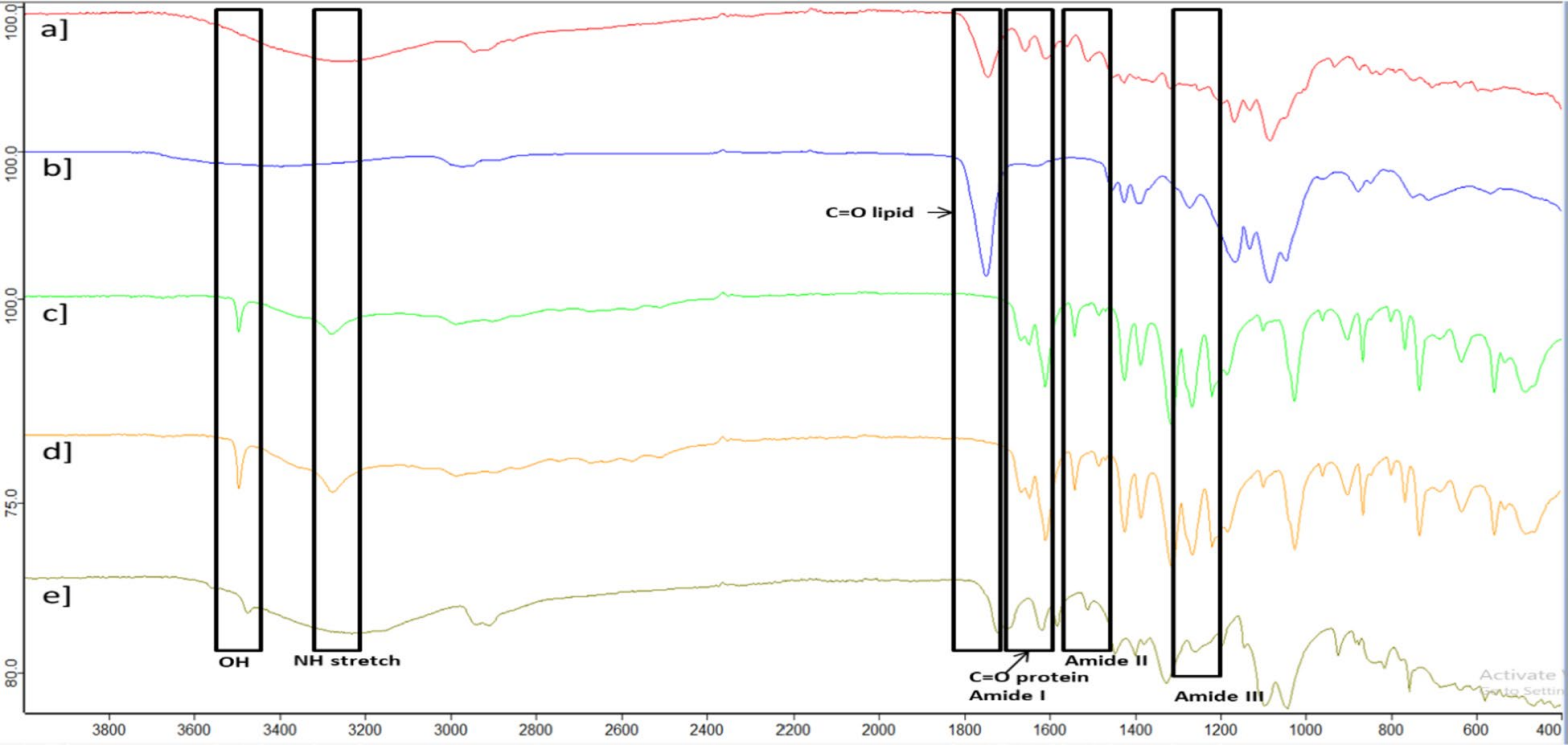
FTIR spectra of PLGA [a], PLGA NPs [b], unloaded PLGA NPs decorated with targeted CD133 antibody [c], and loaded PLGA NPs with Q and K decorated with CD133 antibody [d and e].

The chemical interactions between the ingredients in the loaded PLGA NPs with Q and K decorated with CD133 antibody were assessed using FTIR analysis. The functional groups of the components within the scanning range of 400 to 4000 cm^− 1^ exhibit substantial changes, as represented in Fig. 3. Alterations in the shape, position, and intensity of peaks were indicators of these interactions. PLGA polymer displays characteristic absorption bands at 1100–1250, and 1746–1760 cm^-1^_,_ which represent the esters and carbonyl groups, respectively (Fig. 3a). Also, a hydroxyl group peak can be observed above 3000 cm^-1^. PLGA NPs spectrum depicts characteristic absorption bands at 1746 and 1230–1260 cm^-1^ which express the carbonyl groups and amide III, respectively (Fig. 3b). Either unloaded (Fig. 3c) or loaded (Fig. 3d and e) PLGA NPs decorated with CD133 antibody spectrum depicts characteristic absorption bands at 3450, 3276, 1746, 1630–1660, 1483–1540, and 1230–1260 cm^-1^ which represent the hydroxyl group, NH stretch, carbonyl group (lipid), carbonyl group (protein) with amide I, amide II, an d amide III, respectively.

##### Entrapment efficiency of Q and K within the NP

Entrapment efficiency of Q and K within the NPs decorated with CD133 antibody was assessed using dialysis tubing. The findings revealed that approximately 73% of Q and 83% of K were successfully encapsulated, based on their initial concentrations in the preparations (Table 3).

**Table 3:** Entrapment efficiency of Q and K within the nanoparticle (EE%).

	Quercetin	Kaempferol
Entrapment efficiency (EE%)	73%	83%

##### In vitro release profiles

**Fig. 4:**
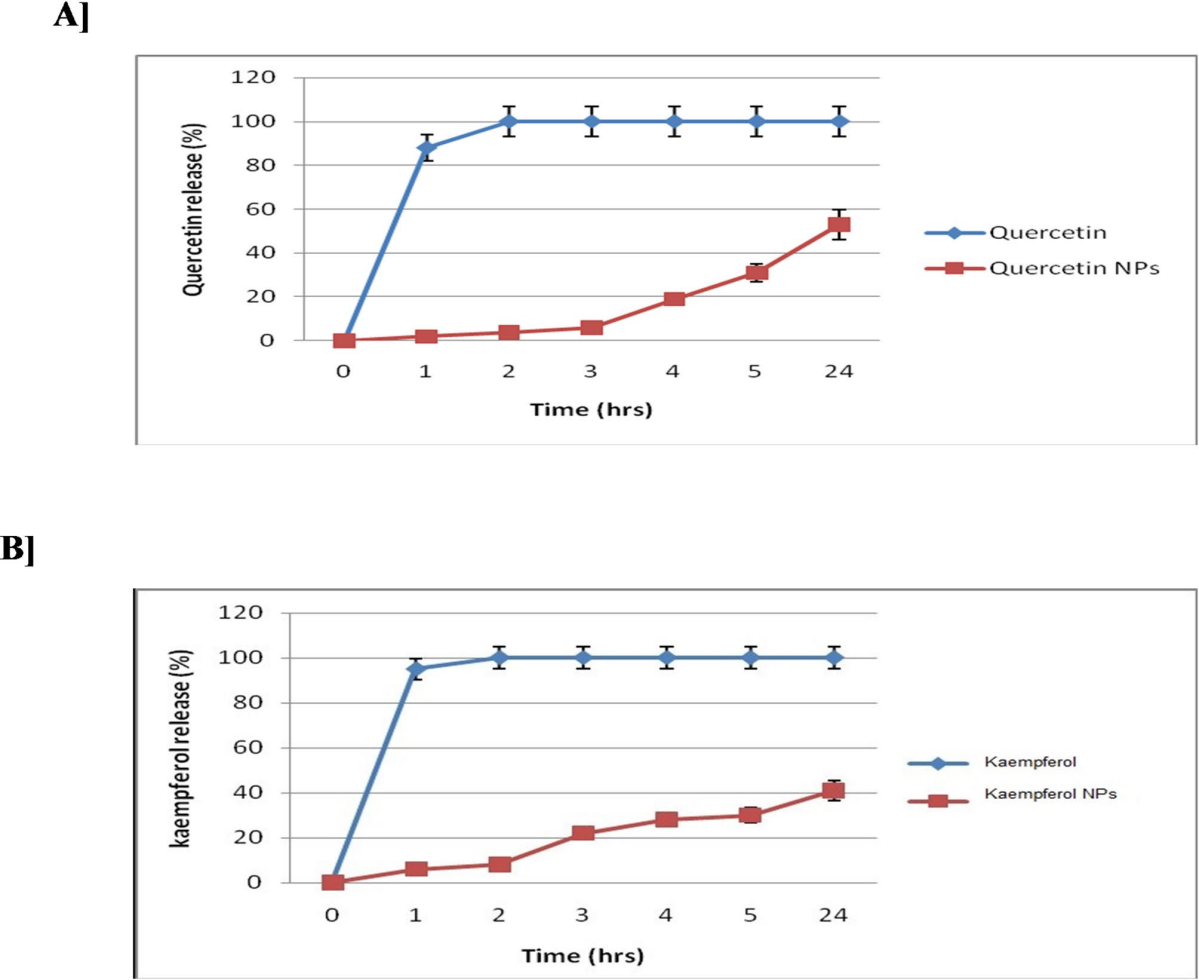
Release profile of free Q and its nanoformula [A] as well as free K and its nanoformula [B] after dialysis against their solvent for 0, 1, 2, 3, 4, 5, and 24 h.

The release profiles of the formulated Q and K NPs with CD133 antibody were assessed at pH 7.4 *versus* the dissolution behavior of the free drugs under identical conditions (Fig. 4). Free Q and K exhibited a rapid burst release, reaching nearly 100% within the first hour. In contrast, the nanoformulations of Q and K showed a more gradual release, with only 2% and 6% released after 1 h, increasing to 53% and 41%, respectively, over 24 h. These findings indicate that the NP system effectively reduces the initial burst release and offers a more controlled and sustained release profile in comparison with the free drug forms.

The drug release kinetic analysis revealed that the release profile of Q from the nanoparticle formulation was best described by the Higuchi model (R² = 0.990), indicating that the release mechanism is primarily diffusion-controlled. The Korsmeyer–Peppas model also showed a good fit, with an exponent *n* < 0.5, further supporting a Fickian diffusion mechanism. Meanwhile, the release profile of K from nanoparticles follows diffusion-controlled kinetics, as indicated by the best fit to the Higuchi model (R² = 0.988). The Korsmeyer–Peppas model also shows a good fit (R² = 0.976) with a diffusion exponent *n* < 0.5, suggesting a Fickian diffusion mechanism. In contrast, free Q and K showed immediate and complete release (~ 100% within 1 h), indicating no controlled release behavior.

#### Characterization of the isolated cells

##### Flow cytometric analysis of the isolated cells from the Huh7 cell line

**Fig. 5:**
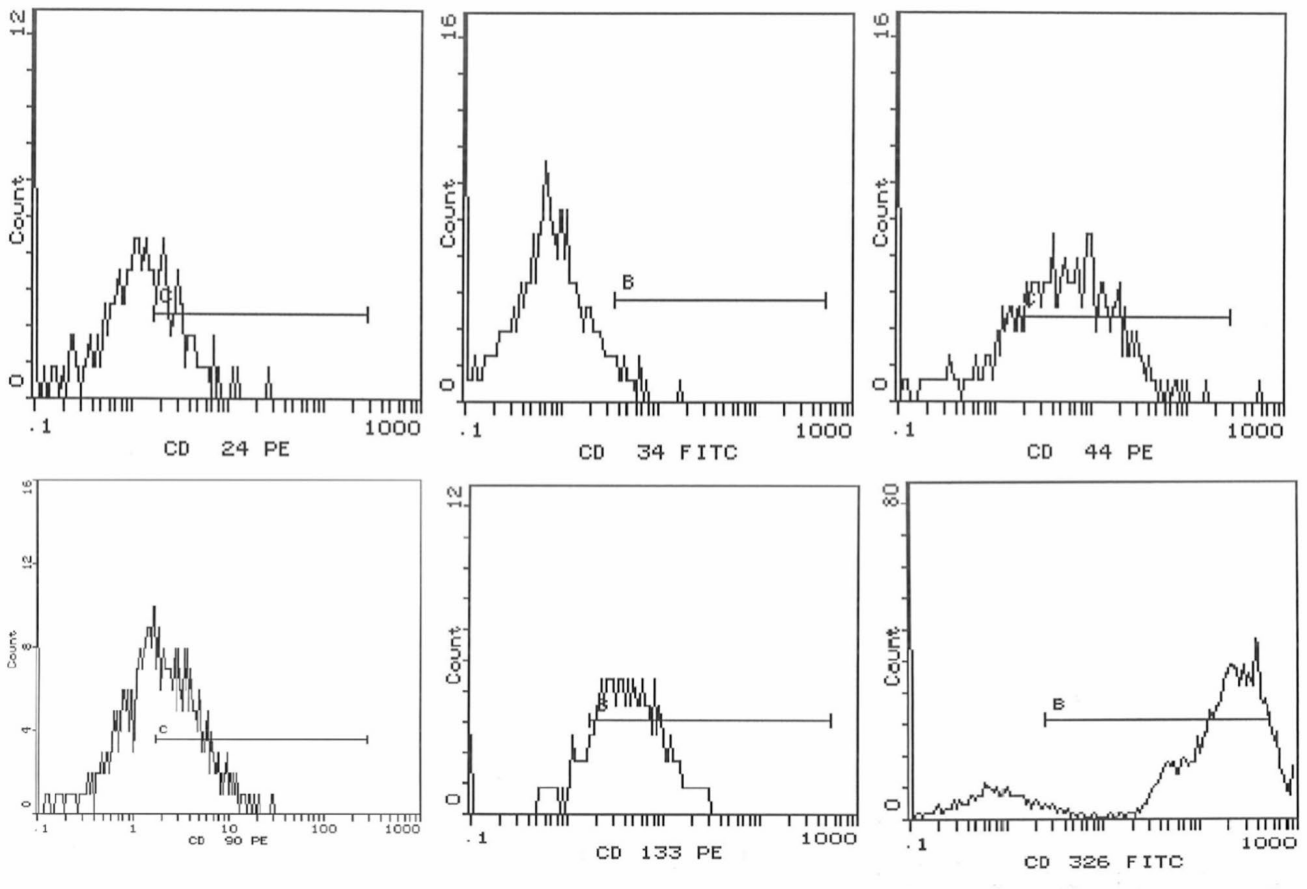
Flow cytometric analysis of the isolated cells from the Huh7 cell line after staining with CD24, CD34, CD44, CD90, CD133, and CD326 antibodies.

Flow cytometric analysis of the isolated cells from the Huh7 cell line revealed positive expression for CD24 (34.1%), CD34 (6.43%), CD44 (70.4%), CD90 (49.6%), CD133 (82.1%), and CD326 (64.4%) (Fig. 5).

##### Expression of CSC markers in the isolated cells from the Huh7 cell line

**Fig. 6:**
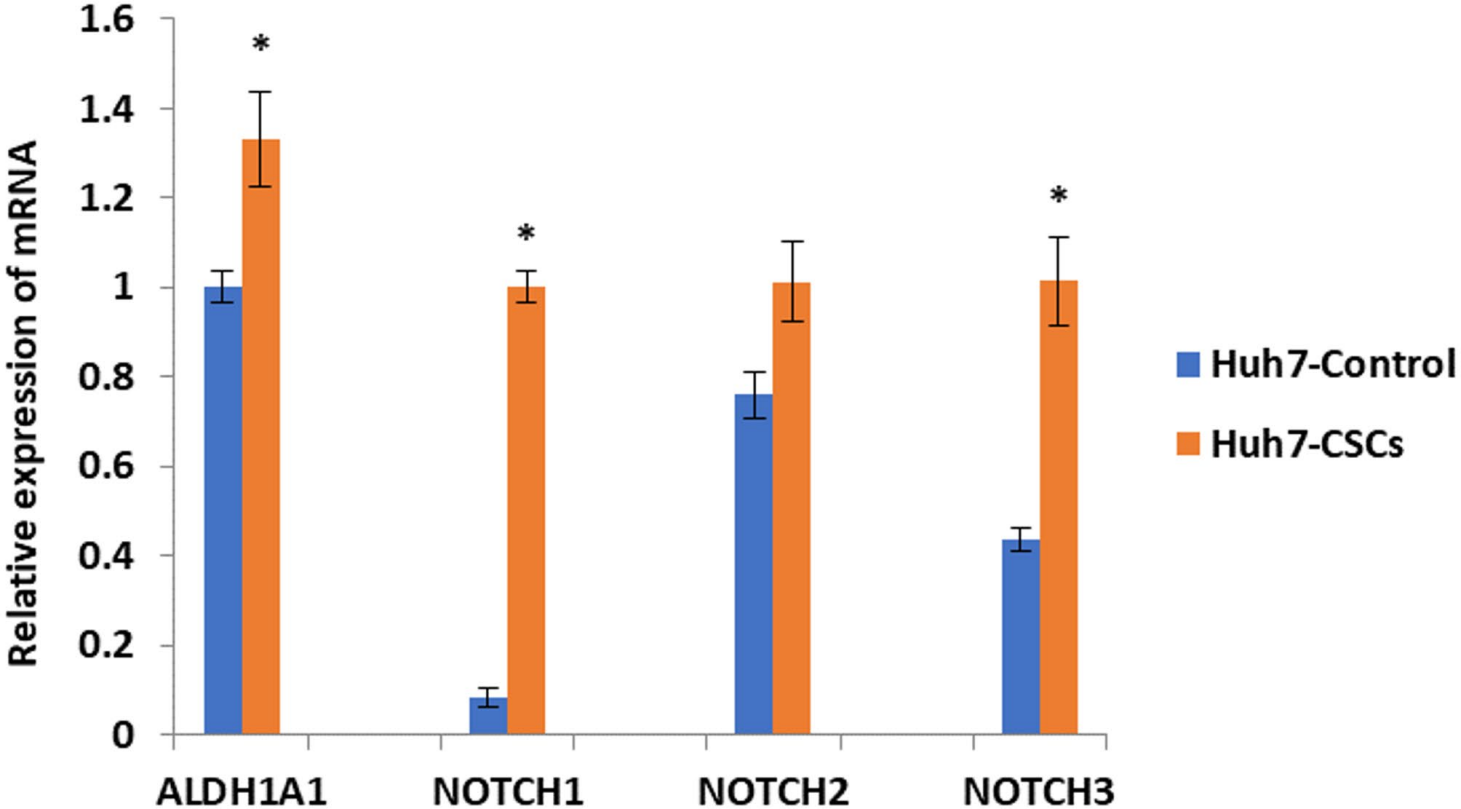
Expression of CSC-related genes in the isolated cells from Huh7 cell lines. All experiments were repeated 3 times. Data are the mean ± SD. * *P* < 0.05 compared with corresponding control cells.

To further characterize the isolated CSCs, real-time PCR analysis was conducted to detect the expression levels of various CSC-related genes. The results demonstrated a significant (*P* < 0.05) upregulation of ALDH1A1, NOTCH1, and NOTCH3 mRNA expression in cells isolated from the Huh7 cell line compared to control cells (Fig. 6). In contrast, the expression of NOTCH2 showed an upregulation that was not statistically significant (*P* > 0.05) relative to the control (Fig. 6).

##### Resistance of the isolated cells from Huh7 cell lines to chemotherapy

**Fig. 7:**
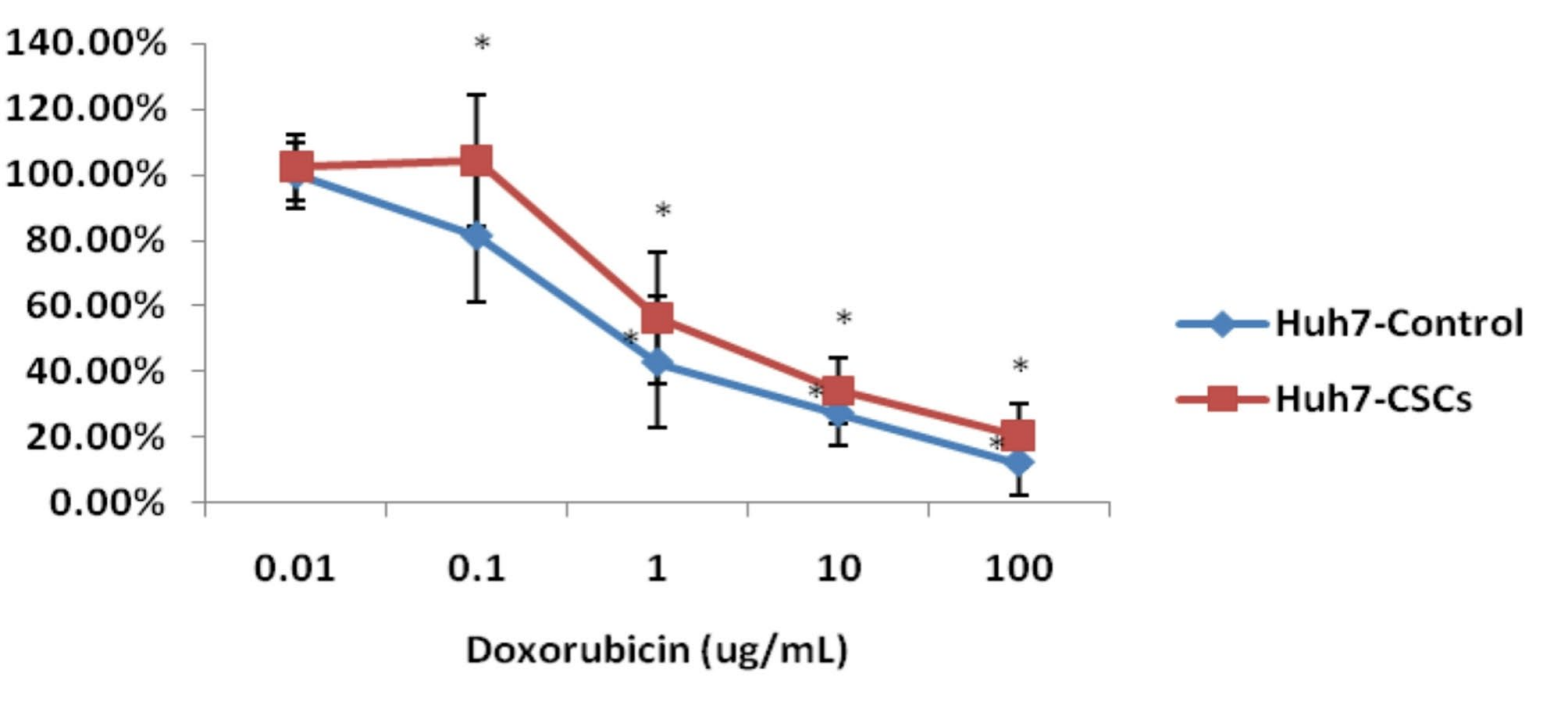
Resistance of the isolated cells to doxorubicin. Data are the mean ± SD. * *P* < 0.05 compared with corresponding control cells.

To assess the potential chemoresistance of the isolated cells from the Huh7 cell line, their sensitivity to different concentrations of doxorubicin was compared with that of control cells. The isolated cells exhibited markedly higher resistance to doxorubicin in comparison with control cells (Fig. 7).

#### Anti-cancer potential of Q and K NPs decorated with CD133 antibody

##### In vitro evaluation of the cytotoxic efficacy of the Q and K NPs decorated with CD133 antibody against hepatic CSCs

**Fig. 8:**
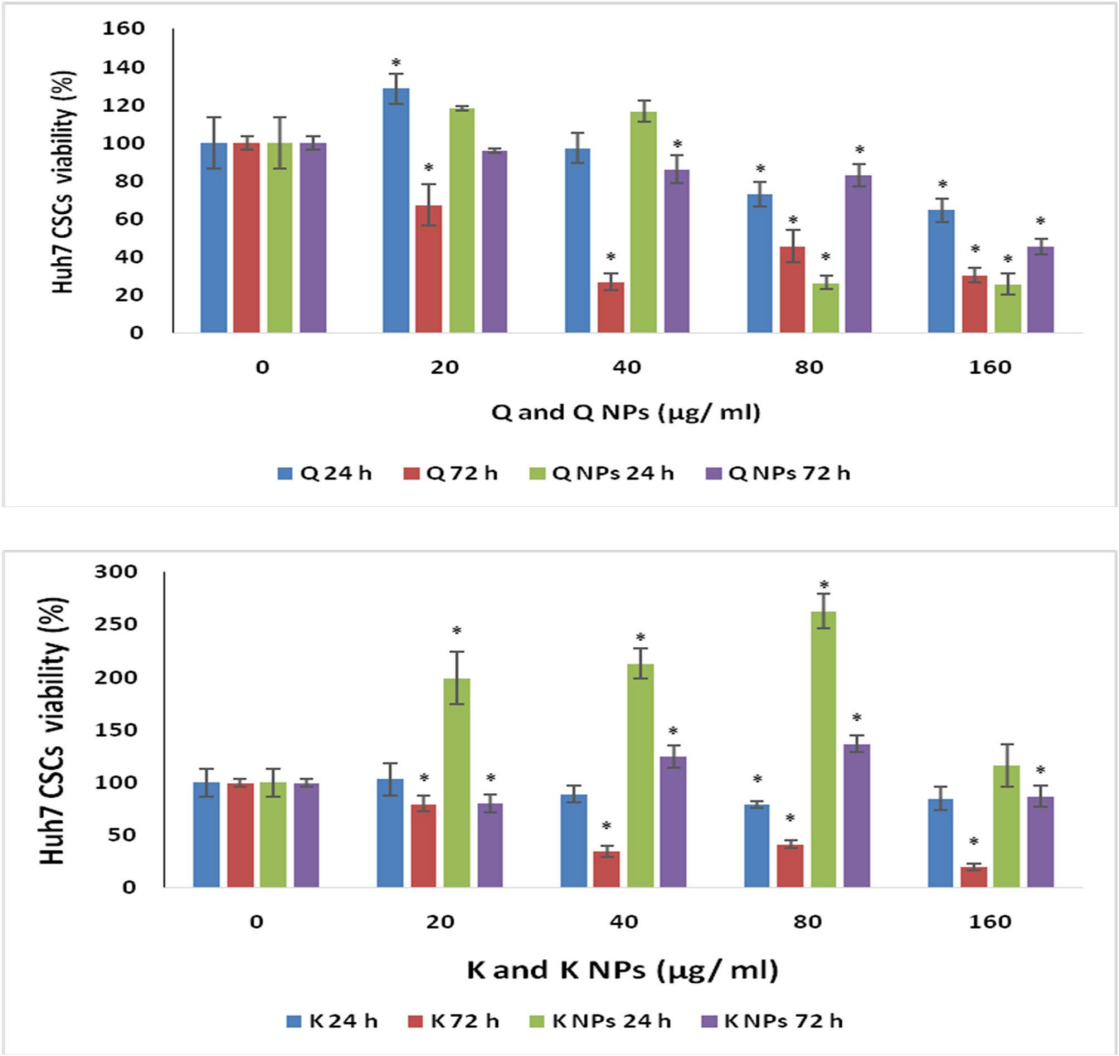
Cytotoxic impact of Q and K NPs and their free forms on CSCs isolated from Huh7 cell line after 24 and 72 h (results are mean ± SD of the triplicate experiments). * Significant change at *P* < 0.05 in comparison with control.

Figure 8 illustrates the cytotoxic effects of a broad concentration range (0–160 µg/ml) of both Q and K NPs and their free forms on CSCs isolated from the Huh7 cell line after 24 and 72 h of treatment. Notably, Q and its NP formulation exhibited significant (*P* < 0.05) cytotoxicity at concentrations of 40 µg/ml and 160 µg/ml, respectively, after 72 h, compared to control cells. In contrast, K and its NP form showed cytotoxic effects at 160 µg/ml and 20 µg/ml, respectively.

##### Apoptotic activity of Q and K NPs decorated with CD133 antibody against hepatic CSCs

**Fig. 9:**
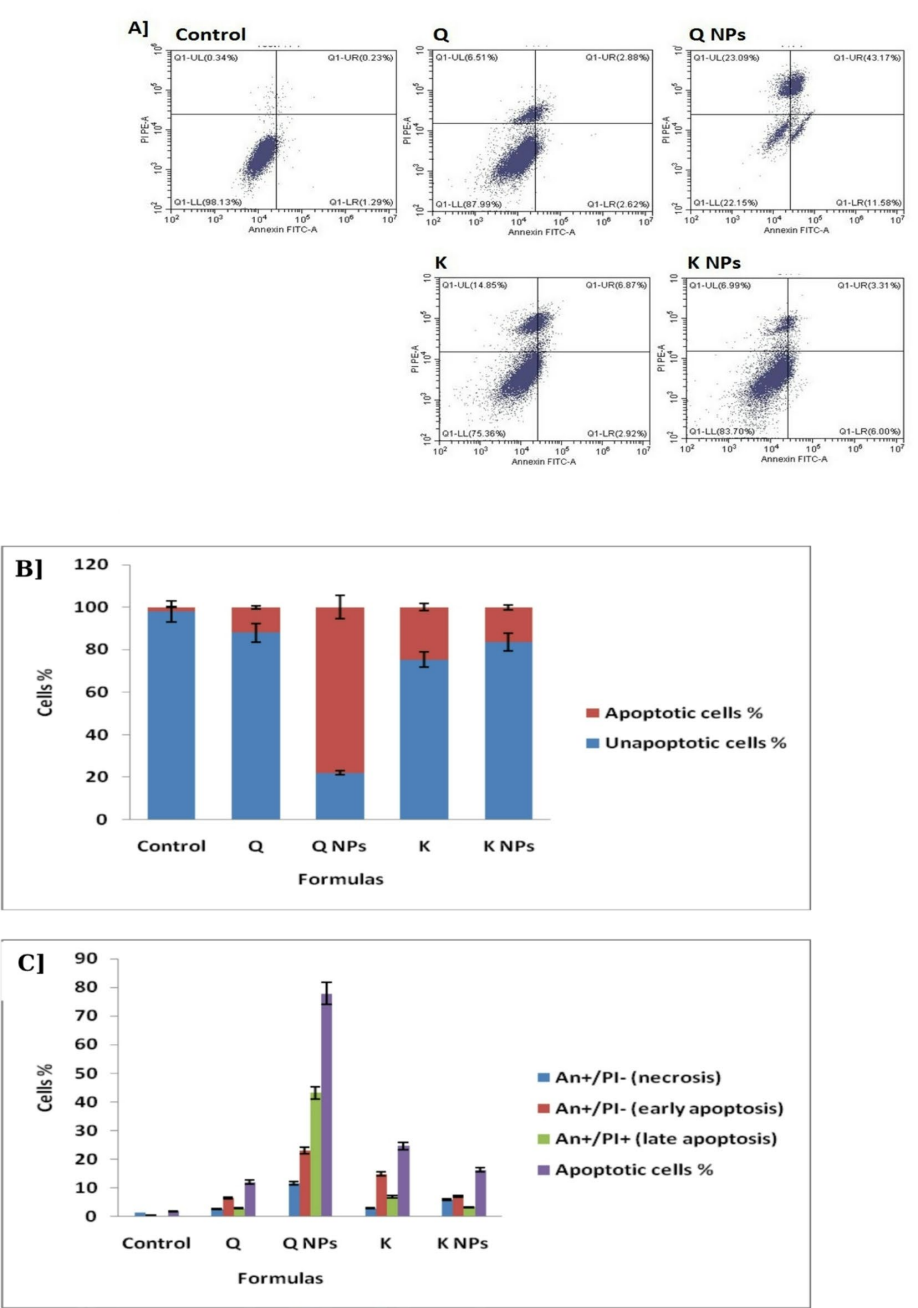
Apoptosis using flow cytometry of the proposed formulas. A] Schematic representation of quadrants for all treatments *versus* control. B] Graph of apoptotic *versus* unapoptotic cells upon treatments. C] Graph of necrotic, early apoptotic, and late apoptotic cells upon treatments.

Following the evaluation of the cytotoxic effect of free Q and free K, along with their nanoformulations (Q NPs and K NPs), against hepatic CSCs, this section explores the mechanistic approach of killing CSCs isolated from Huh7 cell lines. Flow cytometric analysis was employed for this purpose, as it is a widely used technique for distinguishing between necrosis, early apoptosis, and late apoptosis. This method allows for the assessment of individual cells within a population, providing more precise insights. As illustrated in Fig. 9, the percentage of cells in the Annexin V-negative/PI-negative quadrant (indicating viable cells) decreased to varying degrees following treatment with free Q and free K, and more prominently with their respective nanoformulations; Q NPs and K NPs.

Q NPs demonstrated the highest level of apoptosis induction, reaching 77.8%, compared to only 1.8% in the control—a highly significant increase. Detailed flow cytometric analysis revealed that Q NPs primarily induced late apoptosis (Annexin V-positive/PI-positive) in 43.17% of cells, followed by early apoptosis (Annexin V-positive/PI-negative) in 23.09%, along with an 11.58% increase in necrotic cells (Annexin V-negative/PI-positive). When compared to free Q, which induced apoptosis in only 12.01% of cells, Q NPs showed a markedly higher and statistically significant apoptotic effect. Interestingly, cell death induced by free Q occurred predominantly through early apoptosis, whereas Q NPs triggered a stronger late apoptotic response.

K and its nanoformulation induced apoptosis predominantly *via* early apoptotic pathways (Annexin V-positive/PI-negative). K NPs triggered an overall apoptotic response of 16.3%, which, significantly higher than the control (1.8%), while it was notably lower than that observed with Q NPs (77.8%). Specifically, K NPs induced 6.99% early apoptotic cells, 3.31% late apoptotic cells (Annexin V-positive/PI-positive), and 6.0% necrotic cells (Annexin V-negative/PI-positive). When compared to free K, which induced 24.64% total apoptosis, K NPs showed a slightly lower apoptotic effect that was not statistically significant. It is worth noting that free K primarily induced early apoptosis (14.85%), with only a small portion of the cell population progressing to late apoptosis—an essential step for effective CSCs eradication.

##### The inhibitory effect of the Q and K NPs decorated with CD133 antibody on the migration of hepatic CSCs

**Fig. 10:**
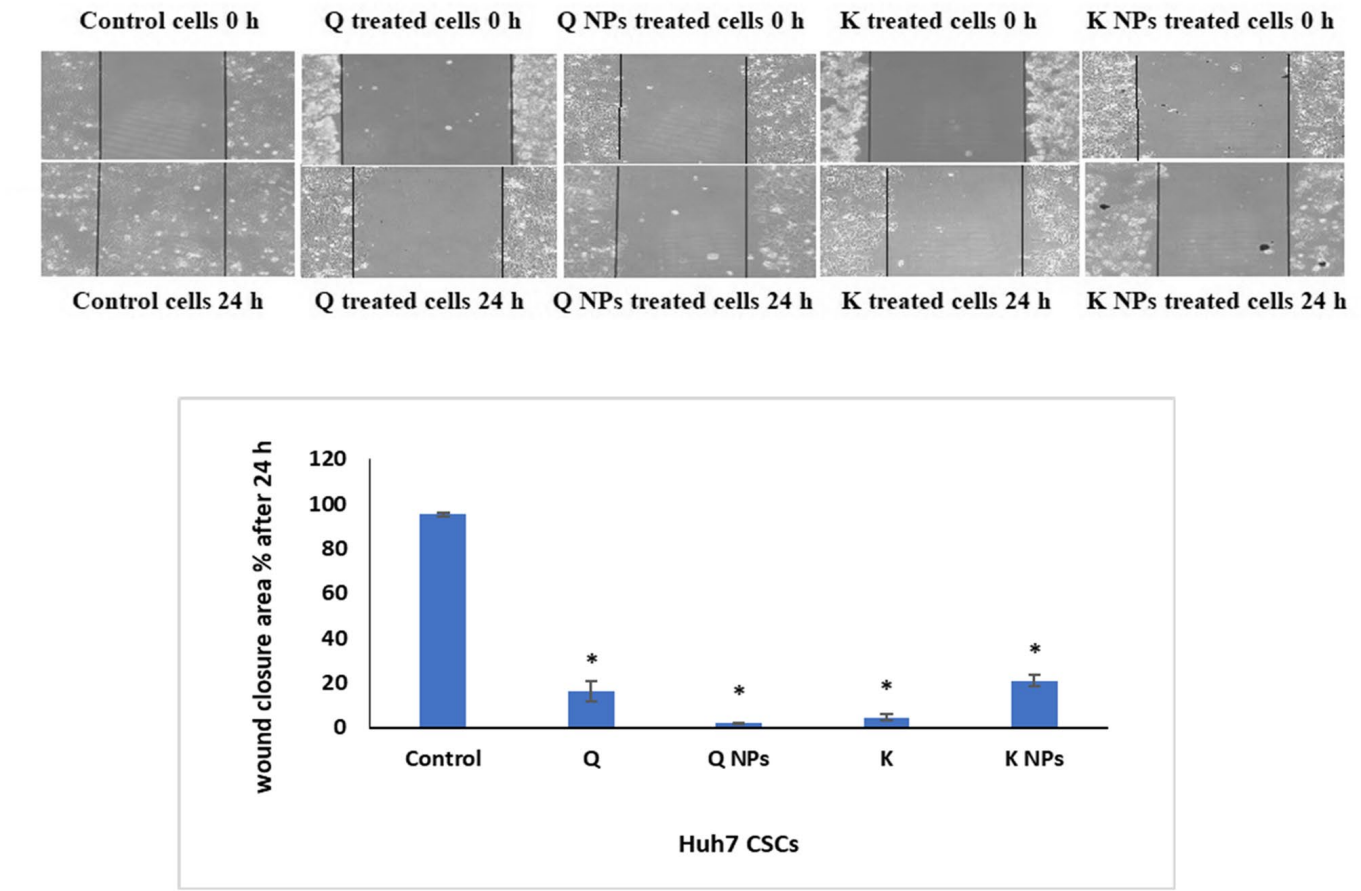
Wound healing assay for demonstrating the inhibitory effect of the Q and K NPs and their free forms on the migration of hepatic CSCs at 24 h following wounding.

The inhibitory effects of Q and K NPs and their free forms on the migration of CSCs isolated from the Huh7 cell line were assessed in vitro using the scratch wound-healing assay. As shown in Fig. 10, all treatments, including both free compounds and their nanoparticle formulations, effectively suppressed the migration of hepatic CSCs as indicated by the significant (*P* < 0.05) decrease in the wound closure area % after 24 h relative to control cells. Notably, the most pronounced inhibition was observed with Q NPs, indicating a strong anti-migratory potential.

##### Gene expression levels of hepatic CSCs-related molecular pathways

**Fig. 11:**
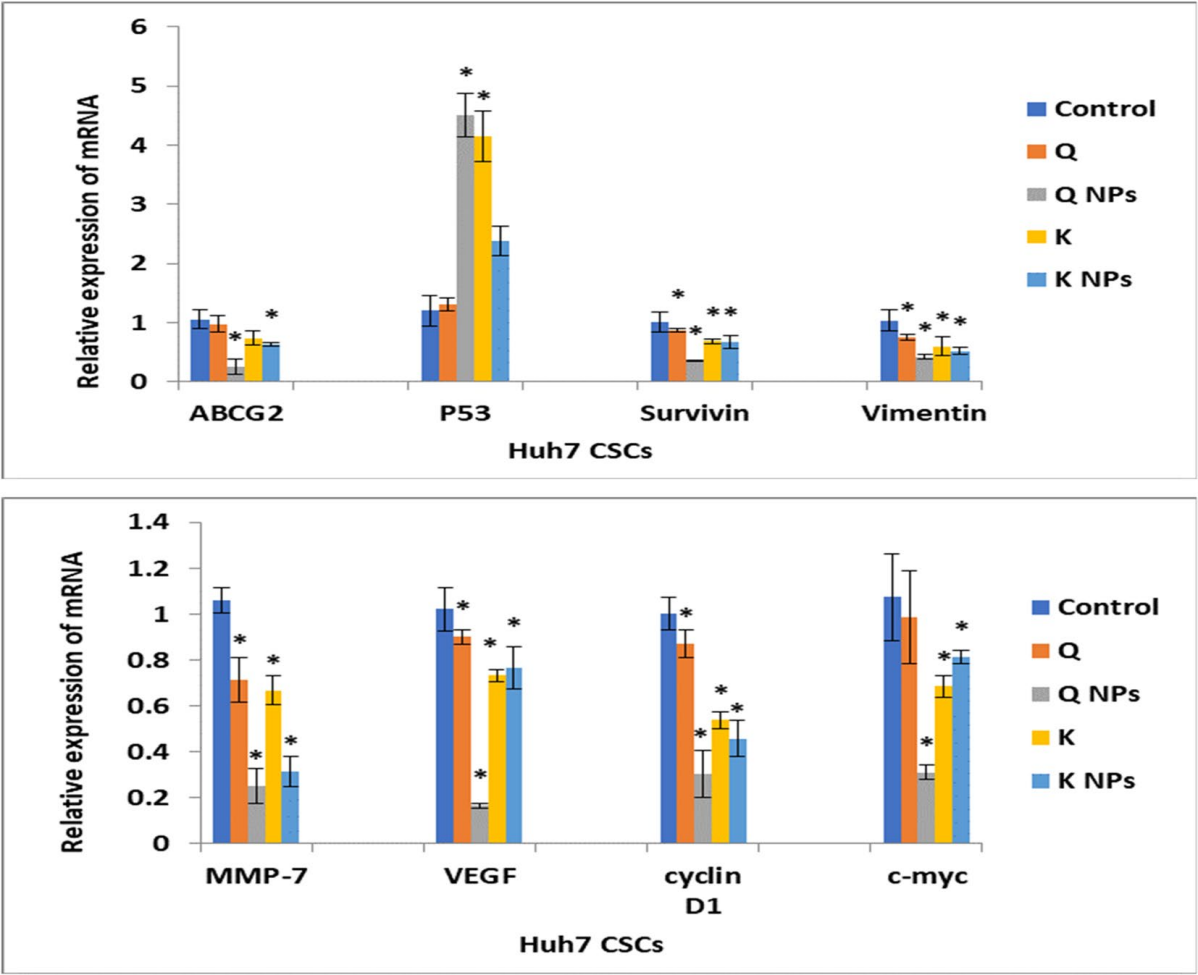
Influence of the Q and K NPs decorated with CD133 antibody and their free forms on the mRNA expression level of ABCG2, P53, survivin, vimentin, MMP-7, VEGF, cyclin D1, and c-Myc in Huh7 CSCs. Data are depicted as mean ± SD, Data were reproducible.  *: Significant change at *P* < 0.05 compared with control cells.

Figure 11 illustrates the influence of Q and K NPs decorated with CD133 antibody and their free forms on the expression levels of several key genes in hepatic CSCs, including ABCG2, P53, survivin, vimentin, cyclin D1, c-Myc, MMP-7, and VEGF. The results confirmed that the treatment with Q NPs and K NPs led to a significant (*P* < 0.05) downregulation of ABCG2 gene expression level compared to the control cells. Additionally, all treatments exhibited a significant (*P* < 0.05) downregulation in the gene expression levels of survivin, vimentin, cyclin D1, MMP-7, and VEGF. Notably, all treatments—except free Q—significantly (*P* < 0.05) downregulated c-Myc gene expression level. In contrast, Q NPs and free K significantly (*P* < 0.05) upregulated P53 gene expression level, suggesting a potential role in promoting apoptosis and tumor suppression.

## Discussion

The theory of CSCs has marked a novel milestone in cancer research, as CSCs are believed to play a key role in tumor recurrence, metastasis, and resistance to chemotherapy^59^. Traditional stemness markers such as Nanog, SRY-box transcription factor 2 (SOX2), and Oct4 are commonly used to identify stem-like properties. In liver cancer, specific CSC markers include CD13, CD24, CD44, CD47, CD90, CD133, intercellular adhesion molecule 1 (ICAM1), EpCAM, and leucine-rich repeat-containing G protein-coupled receptor 5 (LGR5)^60^. In the present study, cells isolated from the Huh7 cell line were found to express CD24 (34.1%), CD34 (6.43%), CD44 (70.4%), CD90 (49.6%), CD133 (82.1%), and CD326 (64.4%). These results fit with those of Chen et al.^61^, who suggested that cells co-expressing CD133 and EpCAM (CD326) are more indicative of CSCs within the Huh7 cell population. Moreover, hepatic CSCs have been reported to predominantly express CD44, which plays a crucial role in facilitating epithelial-mesenchymal transition (EMT)^62^. Furthermore, a previous study investigating the link between liver CSCs and early recurrence of HCC revealed that early recurrence was associated with the expression of CD90^63^. Meanwhile, it has been documented that CD24⁺ hepatoma cells also express CD133 and CD326, with CD24 expression overlapping with these markers. This overlap suggests that CD24, CD133, and CD326 may share similar self-renewal characteristics, highlighting their potential role in identifying cancer stem-like cells^64^.

The current study revealed that hepatic CSCs isolated from the Huh7 cell line showed significant upregulation in the transcriptional levels of NOTCH1, NOTCH3, and ALDH1A, while NOTCH2 exhibited a non-significant increase. The Notch signaling pathway, known for being evolutionarily conserved, plays a critical role in regulating CSC proliferation, self-renewal, differentiation, angiogenesis, and migration^65^. More importantly, the Notch signaling pathway has been reported to be a key player in the acquisition of EMT, which is critically linked to drug resistance. This suggests that targeting the Notch pathway could offer a therapeutic approach for treating cancer by overcoming drug resistance in cancer cells. Such an approach may help eliminate CSCs or EMT-like cells, which are believed to be responsible for tumor relapse^66^. It has been found that NOTCH2 and JAGGED1 are significantly upregulated in gemcitabine-resistant pancreatic cancer cells. Notch signaling downregulation resulted in a partial reversal of the EMT phenotype, inducing a mesenchymal-to-epithelial transition (MET). This transition was associated with a decrease in the expression of vimentin, zinc finger E-box-binding homeobox 1 (ZEB1), Slug, Snail, and NF-κB expression, all of which are key regulators of the EMT process^67^. Persistent activation of the Notch signaling pathway has been suggested to play a crucial role in both reprogramming hepatic progenitor cells (HPCs) into CSCs and in maintaining CSC characteristics throughout HCC development^68^. Luo et al.^69^ indicated that the Notch pathway is turned on in CD90⁺ liver CSCs, suggesting that the Notch pathway may participate in HCC carcinogenesis. Additionally, it has been reported that ABCG2-positive glioma stem cells exhibit high chemoresistance and preferentially express the NOTCH1 gene^70^. In HCC models, ablation of NOTCH3 has been shown to exacerbate the apoptotic response to doxorubicin, a process that is largely dependent on p53^71^. High expression of NOTCH2 has been observed in HCC cells that express CD90^72^. Functional studies have revealed that knockdown of NOTCH2 leads to reduced cell proliferation, impaired cell cycle progression, and decreased colony formation in HepG2 cells. These findings suggest that NOTCH2 is essential for the proliferation and self-renewal of HCC cells^73^.

Earlier studies have shown that cancer cells with high ALDH activity exhibit enhanced tumorigenic capacity and chemoresistance across different cancer types. Specifically, a subpopulation of cells with high ALDH activity has been identified in highly tumorigenic colon CSCs, which display a stem-like EpCAM⁺/CD44⁺ phenotype^74^. Moreover, in breast CSCs, the high ALDH activity has been correlated with more aggressive tumor behavior and increased chemoresistance. As a result, it may serve as a marker for poor clinical outcomes^75^. Ma et al.^76^ have observed that ALDH-positive cells highly express the primitive cell surface marker CD133, suggesting that ALDH could serve as a positive marker for tumorigenic HCC CSCs. These investigators found that CD133⁺ALDH⁺ cells are more tumorigenic than CD133⁺ALDH⁻ cells when grafted into mice. Given these findings, it is possible to propose that ALDH may act as a novel marker of poor prognosis and a potential target for HCC therapy. Additionally, silencing Snail expression significantly downregulates ALDH1 expression, thereby suppressing the stem-like characteristics and in vivo tumorigenic activity of CD44⁺CD24⁻ALDH1⁺ cells^77^.

Following the molecular genetic findings, the isolated cells from the Huh7 cell line in the current study showed a much higher resistance to doxorubicin. This observation aligns with the results of Ma et al.^78^. Growing evidence indicates that the CSC population plays a key role in chemoresistance and cancer relapse, as it has the ability to self-renew and differentiate into various cancer cell lineages in response to chemotherapy^79^. CSCs are also able to trigger cell cycle arrest (a quiescent state), which contributes to their resistance to chemotherapy and radiotherapy^80^. ATP-binding cassette (ABC) transporters are able to release a wide variety of toxin-producing materials from cells, thereby reducing the effectiveness of drugs in killing cancer cells and directly contributing to the development of drug resistance. It has been reported that CSCs overexpress ABC transporters and dysregulate signaling pathway networks, which together contribute to the acquisition of multidrug resistance and the maintenance of self-renewal characteristics^81^.

The dysregulation of Notch, TGF-β, and Wnt/β-catenin signaling pathways has been reported to play a key role in the evolution of CSCs, contributing to the acquisition and maintenance of stem-like traits such as self-renewal, plasticity, and quiescence^60^. Cancer stem cells can be effectively eliminated by targeting core signaling pathways. Given the crosstalk between CSCs and HCC cells, complex CSC niches play a crucial role in tumor growth and resistance to treatment. Single or combination therapies that target these CSC niches have shown effectiveness in treating CSCs. Specifically, targeting CSC surface markers offers a direct approach to addressing these cells^82^.

In the current study, Q and K PLGA NPs functionalized with CD133 antibody were developed to specifically target hepatic CSCs expressing CD133 antigen. The average particle size of the Q NPs with CD133 antibody was approximately 227 nm, with a negative zeta potential of -14.5 mV. The K NPs with CD133 antibody had an average particle size of around 336.4 nm and a negative zeta potential of -15.9 mV. These findings are consistent with a previous study that reported salinomycin CD133-NPs with a suitable size of 149.2 nm and sustained drug release, exhibiting a negative zeta potential of -22.8 mV, similar to our Q NPs with CD133 antibody^83^.

The chemical interactions between the components in the loaded PLGA NPs with Q or K, decorated with the targeted CD133 antibody, were investigated using FTIR scanning. The alterations in the shape, position, and intensity of the peaks served as indicators of these interactions^84^. The PLGA polymer exhibits characteristic absorption bands in the range of 1100–1250 cm⁻¹, corresponding to ester groups, and at 1746–1760 cm⁻¹, representing carbonyl (C = O) groups. Additionally, a broad peak indicating the presence of hydroxyl (–OH) groups can be observed above 3000 cm⁻¹^85,86^. Furthermore, the FTIR spectrum of PLGA NPs shows characteristic absorption bands at 1746 cm⁻¹ and 1230–1260 cm⁻¹, corresponding to carbonyl groups and Amide III, respectively. In both unloaded and drug-loaded PLGA NPs decorated with the targeted CD133 antibody, the spectrum displays distinct absorption bands at 3450 cm⁻¹ (hydroxyl group), 3276 cm⁻¹ (N–H stretch), 1746 cm⁻¹ (carbonyl group—lipid), 1630–1660 cm⁻¹ (carbonyl group—protein/Amide I), 1483–1540 cm⁻¹ (Amide II), and 1230–1260 cm⁻¹ (Amide III)^86,87^. These bands confirm the presence of specific functional groups and successful decoration with the antibody.

The entrapment efficiency of the Q NPs and K NPs with CD133 antibody coupled to their surfaces was approximately 73% and 83%, respectively. Notably, the entrapment efficiency of our formulations was higher than that reported for salinomycin CD133-NPs, which had an efficiency of 63.2%^83^. The nanoformulations of Q and K showed a more gradual release, with only 2% and 6% released after 1 h, increasing to 53% and 41%, respectively, over 24 h. These findings indicate that the NP system effectively reduces the initial burst release and offers a more controlled and sustained release profile in comparison with the free drug forms. The burst release could be attributed to the drug adsorbed onto or close to the surface of the nanoparticles, and also due to the high surface-to-volume ratio of the nanoparticle^88^.

The formulated NPs were further assessed for their potential to induce cytotoxicity, promote apoptosis, inhibit metastasis, reverse drug resistance, and interfere with CSC-associated signaling pathways in Huh7 CSCs, in comparison to their free forms. The results revealed that both Q and Q-loaded NPs with CD133 antibody exerted significant cytotoxic effects on Huh7 CSCs at concentrations of 40 µg/ml and 160 µg/ml, respectively, after 72 h. Notably, Q NPs with CD133 antibody showed the most pronounced apoptotic effect, inducing apoptosis in 77.8% of cells. Specifically, Q NPs with CD133 antibody primarily triggered late apoptosis (43.17%), followed by early apoptosis (23.09%), along with an 11.58% increase in necrotic cells. In contrast, free Q induced cell death predominantly through early apoptosis rather than late apoptosis, as seen with Q NPs with CD133 antibody. Hisaka et al.^89^ recorded that quercetin has been shown to suppress liver cancer cell proliferation *via* promoting apoptosis and causing cell cycle arrest. Studies have reported that Q promotes apoptosis through the upregulation of the tumor suppressor protein p53 and the downregulation of cyclin D1. Additionally, it has been observed to induce cell cycle arrest at the S phase, further contributing to its antiproliferative effects^90^. Q also exerts its antiproliferative and pro-apoptotic effects *via* promoting cell cycle arrest and apoptosis through the phosphorylation of ERK and JNK within the MAPK signaling pathway. Furthermore, it disrupts the PI3K/AKT pathway by promoting the phosphorylation of PI3K, AKT, and S6K, enhancing its anti-cancer activity. Q modulates the Wnt/β-catenin pathway by prohibiting β-catenin nuclear translocation and inhibiting the production of MMPs, which are essential for tumor cell migration. Additionally, it suppresses the JAK/STAT pathway by blocking the formation of phosphorylated STAT (p-STAT), reinforcing its antiproliferative properties. Notably, treatment with Q NPs decorated with CD133 antibody has been shown to effectively inhibit liver cancer cell proliferation, migration, and colony formation, thereby significantly repressing the progression of liver cancer^91^.

The cytotoxic effects of K and its PLGA nanoformulation with CD133 antibody against Huh7 CSCs were evident at concentrations of 160 µg/ml for free K and 20 µg/ml for K NPs with CD133 antibody. Both K and K NPs with CD133 antibody primarily induced apoptosis through early apoptotic mechanisms. Specifically, K NPs with CD133 antibody led to 6.99% early apoptotic cells, followed by 3.31% late apoptosis, and a 6.0% increase in necrotic cells. Overall, K NPs with CD133 antibody induced apoptosis in 16.3% of cells, which was slightly lower and not statistically significant compared to the 24.64% observed with free K. Notably, free K induced cell death mainly through early apoptosis (14.85%), with only a small portion of the cell population advancing to late apoptosis—an important phase for ensuring complete elimination of CSCs. Mylonis et al.^92^ registered that K treatment of Huh7 cells resulted in a marked reduction in cell viability, with this effect being significantly more pronounced under hypoxic conditions. Additionally, K was found to induce G2/M phase cell cycle arrest, accompanied by a decrease in the expression levels of cyclin B and CDK1 in treated cancer cells. Furthermore, K treatment led to a concentration-dependent increase in acidic vesicular organelle (AVO)-positive cells compared to the control group. These findings suggest that K not only impairs cell proliferation but also induces autophagy in Huh7 liver cancer cells^93^. K also contributes to its anticancer activity by activating tumor suppressor genes and inhibiting key pathways involved in cancer progression. These include the suppression of angiogenesis, the PI3K/AKT signaling pathway, STAT3, the transcription factor AP-1, Nrf2, and other critical cell signaling molecules. Through this multifaceted interference, K disrupts cancer cell survival, proliferation, and metastasis^94^.

The wound healing assay results of this study demonstrated that the migration of hepatic CSCs was effectively inhibited by both the proposed NPs with CD133 antibody and their free drug counterparts, with the most significant anti-migratory effect observed following treatment with Q NPs with CD133 antibody. Previous studies have reported that Q promotes the proteasomal degradation of Ras homolog family member C (RhoC), thereby suppressing cell migration and invasion in Huh7 cells. This mechanism likely contributes to the pronounced inhibition of CSCs migration observed in this work^95^. Furthermore, the data from Hung et al.^96^ study suggested that K can suppress the invasion and migration of RCC786-O cells in vitro by downregulating the expression of MMP-2. This effect is achieved through the inhibition of focal adhesion kinase (FAK) and Akt phosphorylation, highlighting K potential to interfere with key signaling pathways involved in cancer cell motility and metastasis. Also, Ju et al.^97^ found that K treatment (25 µM) resulted in a significant reduction in cell migration in both Huh-7 and SK-Hep-1 cells, compared to the vehicle control. This inhibitory effect on migration was attributed to the down-regulation of MMP-9 expression and the inhibition of AKT signaling, which are both crucial for the cell’s migratory and invasive capabilities.

In this study, both Q NPs and K NPs with CD133 antibody led to a marked downregulation of ABCG2 gene expression in Huh7 CSCs. This result is consistent with earlier reports suggesting that phytochemical-based nanoformulations can effectively suppress ABC transporter expression, particularly ABCG2, which is often associated with drug resistance and CSCs maintenance^98,99^. Such downregulation may contribute to overcoming chemoresistance and improving therapeutic outcomes^100^. Breast cancer resistance protein (BCRP, also known as ABCG2) belongs to the ATP-binding cassette G family and plays a significant role in the efflux of various anticancer drugs, thereby contributing to the development of chemoresistance, particularly in digestive system cancers. ABCG2 expression is notably elevated in the side population (SP) of stem-like cells, which exhibit enhanced drug resistance compared to non-stem-like cancer cells, highlighting its importance in maintaining the chemoresistant phenotype of CSCs^101^. Interestingly, ABCG2 expression has been closely associated with key aspects of HCC progression, including tumor initiation, cell proliferation, metastasis, and the development of chemoresistance^102^. Thus, BCRP (ABCG2) serves as a potential marker for liver CSCs^103,104^. Namisaki et al.^105^ recorded that ABCG2 expression, which is subject to modulation by the AKT signaling pathway, has a substantial role in mediating the efflux of chemotherapeutic agents like doxorubicin from HCC cells, thereby reducing their therapeutic effectiveness. Moreover, the emergence of a liver CSCs phenotype—characterized by elevated ABCG2 expression—is strongly linked to malignant behaviors, including enhanced proliferation, migration, and invasion, further highlighting ABCG2’s involvement in HCC aggressiveness and treatment resistance^104^. Notably, these malignant characteristics can be significantly diminished by downregulating ABCG2 expression. This highlights the potential therapeutic value of targeting ABCG2 to impair liver CSC traits and enhance the effectiveness of anticancer treatments in HCC^103^.

Epithelial-mesenchymal transition is recognized as a key mechanism driving chemoresistance in HCC. During this process, HCC cells exhibit downregulation of epithelial markers such as desmoplakin, E-cadherin, and claudin-1, which are essential for maintaining cell-cell adhesion and epithelial integrity. Concurrently, there is an upregulation of mesenchymal markers and EMT-related factors, including neural (N)-cadherin, vimentin, matrix metalloproteinases, and several transcription factors such as Snail1, Snail2, ZEB1, ZEB2, and Twist. This phenotypic shift enhances cell motility, invasiveness, and contributes to drug resistance, ultimately facilitating tumor progression and metastasis^106^. Furthermore, EMT initiates the activation of several key signaling pathways that are pivotal in promoting tumor progression and therapy resistance. These include the transforming growth factor (TGF)-β/SMAD pathway, Wnt/β-catenin signaling, the mitogen-activated protein kinase/extracellular signal-regulated kinase (MAPK/ERK) cascade, the phosphoinositide 3-kinase (PI3K)/protein kinase B (Akt) axis, and the Notch signaling pathway^107^. These EMT-induced alterations markedly enhance the resistance of HCC cells to apoptosis and chemotherapeutic agents. A critical link between angiogenesis and HCC progression lies in the epithelial-to-mesenchymal transition of liver epithelial cells, which is often driven by angiogenic factors—particularly VEGF. VEGF not only promotes neovascularization but also facilitates EMT, thereby accelerating tumor growth, invasion, and resistance to treatment^108^. Furthermore, vimentin filaments play a crucial role in protecting cancer cells from mechanical stresses encountered during migration or when squeezing through confined spaces. By supplying a viscoelastic framework, vimentin ensures the structural integrity of the cell, particularly the positioning and stability of organelles, including the nucleus. This function is essential during EMT and cancer progression, as it helps cancer cells maintain their shape and adapt to the physical challenges of invasion and metastasis^109^.

MMP-7 has been extensively studied in cancer progression due to its dual role in both extracellular matrix degradation and metastasis promotion, as well as its involvement in the regulation of the Fas/FasL system and apoptosis sensitivity in tumor cells. MMP-7 influences Fas expression and activation by cleaving the membrane-bound form of FasL, generating its soluble form^110^, and by cleaving the Fas receptor itself. In both instances, MMP-7 activity effectively blocks the induction of apoptosis by Fas activation. This protective mechanism allows tumor cells to evade apoptosis, contributing to enhanced cell survival, resistance to chemotherapy, and overall tumor progression^111^. Furthermore, survivin has been identified as a downstream gene of the Wnt/β-catenin signaling pathway, which has a fundamental role in the regulation of gastric CSCs. The activation of this pathway promotes the expression of survivin, a protein that inhibits apoptosis and supports cell survival, contributing to the maintenance of CSC properties such as self-renewal, tumorigenicity, and resistance to chemotherapy^112^. It has been recorded that glioma stem cells (GSCs) contribute to treatment resistance in tumor cells by upregulating DNA damage checkpoint proteins^113^. CSCs and survivin are widely recognized as key factors contributing to the recurrence of tumors, and radiation and drug resistance observed in recurrent tumors^114^. On the other hand, TGF-β/Smad signaling plays a critical role as an effector in various signaling pathways regulating the self-renewal of both normal and CSCs. Cyclin D1 serves as a key regulator of early cell fate decisions during the G1 phase of human embryonic stem cells (hESCs) by controlling the transcriptional activity of Smad2/3^115^.

In the current study, the treatment of Huh7 CSCs with Q, Q NPs with CD133 antibody, K, and K NPs with CD133 antibody downregulated the expression levels of VEGF, vimentin, MMP-7, survivin, and cyclin D1 genes. These results are in agreement with those of previous studies, which demonstrated the anti-angiogenic, anti-metastatic, and pro-apoptotic effects of Q^116^ and K^117,118^ in various cancer models. For instance, it has been reported that Q suppresses VEGF expression and angiogenesis by modulating HIF-1α and PI3K/Akt signaling^119^. Furthermore, Wu et al.^120^ clarified that Q has been reported to reverse invasion and migration by modulating EMT-related markers. Specifically, it upregulated E-cadherin—a key epithelial marker—while downregulating mesenchymal markers like N-cadherin and vimentin at both gene and protein expression levels in HCC LM3 cells. This shift from a mesenchymal to an epithelial phenotype suggests that Q can suppress EMT, thereby limiting cancer cell migration, invasiveness, and possibly reducing the stem-like properties associated with cancer progression and resistance. While, Lee et al.^121^ stated that, Q dose-dependently inhibited the transcriptional activity of specificity protein 1 (Sp1) and subsequently downregulated the expression of its downstream targets, including p27, p21, cyclin D1, Mcl-1, and survivin in HepG2 cells. This suggests that Q can interfere with Sp1-mediated transcriptional regulation, leading to cell cycle arrest and apoptosis. In addition, Q NPs were found to upregulate p27, a cyclin-dependent kinase inhibitor that plays a crucial role in inducing cell cycle arrest in liver cancer cells. Moreover, treatment with Q NPs led to the significant, dose-dependent inhibition of several key oncogenic and cell cycle-related proteins, including c-Myc, cyclin-D1, CDK1, MMP-7, and β-catenin^32^. These molecular changes contributed to the induction of apoptosis in liver cancer cells, further supporting the therapeutic potential of Q nanoformulations in targeting liver CSCs and overcoming chemoresistance.

It was shown that K inhibits the expression of VEGF in ovarian cancer cells, thereby reducing angiogenesis, which is crucial for tumor growth and metastasis^122^. Zhu et al.^123^ found that K dramatically suppressed the viability, proliferation, migration, and invasion of HepG2 liver cancer cells. Notably, K treatment led to a significant downregulation in the expression levels of cyclin D1, MMP-2, MMP-9, and vimentin^124,125^. Colombo et al.^126^ demonstrated that, K effectively suppressed glioma cell growth and migration when administered *via* K-loaded nanoemulsion and mucoadhesive nanoemulsion formulations.

c-Myc is indeed a critical “master” transcription factor that orchestrates the expression of a broad spectrum of genes essential for various cellular processes, including cell proliferation, differentiation, angiogenesis, apoptosis, metabolism, EMT, invasion, and metastasis^127^. Absolutely, c-Myc has been shown to regulate the expression of vimentin and associated pathways for the induction of EMT and cell migration^128^. Previous research has demonstrated that the deletion of the tumor suppressor gene p53 promotes hepatocyte proliferation and tumor formation^129^. In addition to the deletion of p53, the Bcl-2 and Bmi-1 overexpression, along with p19ARF loss, further aid Myc in regulating the survival and proliferation of CSCs^130,131^.

In the current investigation, Q NPs with CD133 antibody, K, and K NPs with CD133 antibody were found to significantly downregulate c-Myc expression levels in Huh7 CSCs. Moreover, treatment of Huh7 CSCs with Q NPs with CD133 antibody and K led to a significant upregulation of P53 gene expression. These findings are consistent with the results reported by previous studies, which demonstrated that Q and K can modulate critical oncogenes and tumor suppressor genes involved in cancer progression and stemness maintenance^132,133^. Shahbaz et al.^133^ declared that Q exerted multiple antitumor effects on pancreatic cancer cells, including the apoptosis induction, proliferation inhibition, migration and invasion suppression, and metastasis and tumor growth reduction in pancreatic ductal adenocarcinoma (PDA) xenograft models. These effects are largely attributed to its ability to downregulate c-Myc expression, which significantly limits the proliferative capacity of pancreatic cancer cells, while simultaneously upregulating p53. Furthermore, Zhang and Ma^132^ mentioned that K enhanced the efficacy of cisplatin by modulating key regulatory genes involved in cell cycle control and apoptosis. Specifically, K reduces c-Myc mRNA levels while upregulating cyclin-dependent kinase inhibitor 1 A (CDKN1A), which plays a crucial role in arresting the cell cycle. Interestingly, in breast cancer MDA-MB-453 cells, K appears to enhance p53 expression, suggesting a dual mechanism of action—both suppressing oncogenic signals and promoting tumor suppressor pathways.

In conclusion, the findings of this study spotlight the effective targeting of hepatic CSCs by CD133 antibody surface-modified quercetin and kaempferol NPs. This efficacy was achieved through their capacity to induce apoptosis, inhibit metastasis, overcome drug resistance, and modulate key CSCs-related signaling pathways. Hence, nanoparticle surface modification with CD133 antibody emerges as a highly beneficial strategy for enhancing therapeutic properties of the anti-cancer agents, rendering these NPs multifunctional and particularly well-suited for targeting CSCs in hepatocellular carcinoma.

## Data Availability

The datasets used and/or analyzed during the current study are available from the corresponding author on reasonable request.
